# Therapy Dogs as a Crisis Intervention After Traumatic Events? – An Experimental Study

**DOI:** 10.3389/fpsyg.2018.01627

**Published:** 2018-09-04

**Authors:** Johanna Lass-Hennemann, Sarah K. Schäfer, Sonja Römer, Elena Holz, Markus Streb, Tanja Michael

**Affiliations:** Division of Clinical Psychology and Psychotherapy, Department of Psychology, Saarland University, Saarbrücken, Germany

**Keywords:** PTSD, animal assisted therapy, service dogs, stress, cortisol, trauma film paradigm

## Abstract

Animal-assisted therapy has been proposed as a treatment adjunct for traumatized patients. In animal-assisted crisis response, dogs are used directly after a traumatic event to reduce stress and anxiety. However, to date there are few controlled studies investigating the effects of therapy dogs on PTSD symptoms and to our knowledge there is no study investigating the effects of a therapy dog intervention directly after a traumatic event. In this study, 60 healthy female participants were randomly assigned to one of three groups: after exposure to a “traumatic” film clip (trauma-film paradigm), one group of participants interacted with a friendly dog for 15 min, another group of participants watched a film clip showing a person interacting with a friendly dog and the last group was instructed to relax. Participants who had interacted with the dog after the film reported lower anxiety levels, less negative affect, and more positive affect after the intervention as compared to the other two groups. However, the participants who interacted with the dog showed a smaller decrease in physiological arousal after the traumatic film clip compared to both other groups. There were no differences in intrusion symptoms between the three groups. Our results show that dogs are able to lessen subjectively experienced stress and anxiety after a “traumatic” stress situation.

## Introduction

During the last decade, the integration of animals into psychotherapy and health care has greatly increased. While animal-assisted interventions were originally established for the elderly, disabled persons, and children, in recent years the interest in the use of animal-assisted interventions for traumatized individuals and PTSD patients has grown. Several programs have developed dog-assisted programs for PTSD patients and both the anecdotal evidence as well as the first clinical evidence of these programs are promising (Yount et al., 2012; O’Haire and Rodriguez, 2018). PTSD service dogs seem to reduce stress and anxiety in stressful situations, especially when patients are confronted with situations that remind them of the traumatic event. To date, there are three branches of animal-assisted interventions for traumatized individuals: (1) service dogs for PTSD patients, (2) animal-assisted therapy as a part of/or an adjunct to psychotherapy (Lass-Hennemann et al., 2015) and (3) animal-assisted crisis response (AACR). Animal-assisted crisis response is a relatively new field in animal-assisted interventions, in which trained dog/handler teams provide animal-assisted support to individuals affected by trauma and crisis (Greenbaum, 2006). In detail, trained dog/handler teams are called to sites of crises and provide comfort, stress relief, and emotional support to those affected by crises and disasters. In the United States AACR teams have been active in a variety of crisis sites, including the World Trade Center attack (2001), Hurricane Katrina (2005), the Virginia Tech massacre (2007), the Northern Illinois University shooting (2008), and most recently, the Sandy Hook Elementary shootings (2012). Amongst other effects, AACR teams are believed to act as a calming agent to traumatized individuals. Indeed, a large body of evidence has shown that dogs have anxiety and stress reducing effects. These effects have been shown for physiological and endocrine parameters and for subjective anxiety and stress ratings in stressful situations as well as under resting conditions (for a review see Beetz et al., 2012b). Studies investigating stress parameters under resting conditions have demonstrated that stroking and interacting with dogs reduced cortisol levels and physiological stress markers in healthy adults (Friedmann et al., 1983; Jenkins, 1986; Vormbrock and Grossberg, 1988; Odendaal, 2000; Odendaal and Meintjes, 2003; Barker et al., 2005; Handlin et al., 2011) and children (Friedmann et al., 1983) as well as in physically impaired adults (Cole et al., 2007) and autistic children (Viau et al., 2010). Studies investigating the influence of dogs on subjective and physiological stress parameters in stressful conditions also showed that dogs are able to reduce physiological and endocrine stress responses after cognitive stressors in healthy adults (Allen et al., 1991, 2002; Demello, 1999). Studies in children using mild natural stressors (e.g., physical examinations) and social-cognitive laboratory stressors (e.g., Trier social stress test) found that the presence of a dog reduced endocrine and physiological stress markers as well as subjective stress ratings (Beetz et al., 2012a; Kertes et al., 2017). While a recent review reports that studies find reduced anxiety levels as a response to animal-assisted interventions in PTSD patients (such as therapeutic riding programs) (O’Haire et al., 2015), thus far there is only one study analyzing the effects of animal-assisted interventions to “traumatic” stressors (Lass-Hennemann et al., 2014). In this study, we were able to show that dogs reduce subjective stress ratings in “traumatic” situations. Healthy participants that were accompanied by a friendly dog, while watching a traumatic film (which reliably induces stress, anxiety, and intrusive memories) experienced less anxiety and negative affect after the “traumatic” film clip as compared to control groups.

To summarize, AACR is an intervention intended to reduce stress and anxiety after traumatic situations. Further, research has shown that animals, especially dogs, have stress and anxiety reducing effects. However, to date there is no empirical evidence showing that animals are able to reduce stress through their presence after traumatic situations. Thus, in this study, 60 healthy female participants were exposed to a “traumatic” film clip (trauma-film paradigm) (James et al., 2016) and subsequently randomly allocate to one of three experimental groups. After the film, one group of participants interacted with a friendly dog for 15 min (“dog group”), one group of participants watched a film clip showing a person interacting with a friendly dog (“dog-film group”), and the last group was instructed to relax to control for unspecific, not dog-related effects of simple relaxation (“alone group”). We assessed subjective stress ratings as well as endocrinological and physiological stress responses in reaction to the traumatic film clip as well as intrusive memories regarding the content of the trauma-film. We expected participants in the “dog group” to show lower subjective stress and anxiety ratings following the dog intervention than participants in the control groups. We also expected participants in the “dog group” to show a faster recovery from the physiological arousal caused by the traumatic film than participants in the control groups. Even though there are some studies showing reduced PTSD symptoms after animal-assisted interventions (e.g., Earles et al., 2015; O’Haire and Rodriguez, 2018), there is no study analyzing the effect of animal-assisted interventions on intrusion measures specifically. Thus, we did not have a strong hypothesis concerning the influence of the therapy dog intervention after a “traumatic” event on intrusive reexperiencing symptoms.

## Materials and Methods

### Participants

Participants were 60 healthy female students at Saarland University, Germany, who responded to flyers offering 20 Euro for taking part in a psychological experiment. Exclusion criteria were: fear of dogs, dog hair allergy, life-time traumatic experiences, any axis I mental disorder or current psychotherapeutic treatment, pregnancy and lactating. Due to the assessment of cortisol, participation was restricted to healthy, non-smoking students with a body mass index between 20 and 25 kg/m^2^. Only women with regular use of monophasic oral contraceptives were included to minimize the influence of menstrual cycle phase on hormonal status. Additionally, those taking monophasic contraceptives containing drosperinone (e.g., *Yasmin, Yasminelle*, or *Petibelle)* were also excluded as these may inhibit the endogenous cortisol synthesis (Genazzani et al., 2007). We also required participants to refrain from physical exercise, alcohol and caffeinated drinks 3 h prior to the experimental session. Experimental sessions took place between 2 p.m. and 6 p.m. to control for the diurnal cycle of cortisol. All participants gave written informed consent and the research design was approved by the responsible local ethics committee.

### Materials

#### Trauma-Film

The trauma-film consists of an 11-min compilation of scenes from the film “Irreversible” directed by Gaspar Noe. It contains fictional scenes depicting physical and sexual violence. The trauma-film has been used in previous studies in our laboratory (e.g., Lass-Hennemann et al., 2014; Streb et al., 2016; Graebener et al., 2017) and other laboratories (e.g., Nixon et al., 2009a,b). It has been shown to reliably induce physiological and subjective stress responses as well as intrusive memories. Participants were informed in the study advertisement, participant information, and informed consent process that the film-clip contained graphic material which could be disturbing, and that they were free to withdraw from the study at any time without penalty.

#### Subjective Stress and Anxiety Measurements

##### STAI-S

We used the German version of the state scale of the STAI (Laux et al., 1981) to measure participants’ change in anxiety level in response to the traumatic film clip and the dog intervention. The STAI-S is a brief self-report measure consisting of 20 items related to feelings of apprehension, nervousness, tension, and worry. Participants are asked to rate items on a scale from 1 to 4, where ‘1 = not at all’ and ‘4 = totally agree.’ The score for the STAI-S scale ranges from 20 to 80, with 20 indicating a very low and 80 a very high state-anxiety level. The internal consistency for the state scale of the STAI-S is high.

##### Positive and Negative Affect Schedule

We used the German version of the PANAS (Krohne et al., 1996) to assess changes in positive and negative mood before and after the trauma-film and after the dog intervention. The PANAS questionnaire consists of 10 positive affects (PAs, interested, excited, strong, enthusiastic, proud, alert, inspired, determined, attentive, and active) and 10 negative affects (NAs, distressed, upset, guilty, scared, hostile, irritable, ashamed, nervous, jittery, and afraid). Participants are asked to rate items on a scale from 1 to 5, on the basis of the strength of emotion where ‘1 = very slightly or not at all’ and ‘5 = extremely.’ For both of the PANAS domains, scores can range between 10 and 50. A higher score on the positive domain indicates greater PA, a higher score on the negative domain greater NA. The PANAS has been shown to be a reliable and valid questionnaire. Crawford and Henry (2004) showed that higher NA is linked to anxiety and lower PA to depression symptoms.

#### Intrusion and Thought Frequency and Distress

Participants were asked to record intrusion frequency and thought frequency as well as their distress during the 4 days following the experimental session. Intrusions were defined as “sudden, spontaneous and non-initiated memories of film scenes that might be very vivid and consist of pictures, sounds, thoughts, words or sentences, feelings or combinations of those” (translated from German). Furthermore, participants were asked to record all thoughts relating to the traumatic film clip. Thoughts and intrusions were clearly differentiated in advance by the following instruction: “Intrusions do not include reflective and conscious thinking or ruminating about the film” (translated from German). Participants were asked to only report intrusions and thoughts that were related to the trauma-film. After 4 days participants received an online-questionnaire, in which they were asked to type in the number of intrusions, intrusion distress [on a scale from ‘not at all’ to ‘extremely’ (0–10)], the number of thoughts and thought distress [on a scale from ‘not at all’ to ‘extremely’ (0–10)].

#### Physiological Stress Measurements

##### Blood pressure (BP)

Systolic and diastolic blood pressure was measured using a DINAMAP V100 device (GE-Healthcare, Munich, Germany) with a cuff placed around the upper arm. Blood pressure was measured six times [before the film, during the film (after 5.30 min had passed) and after the film (start of dog intervention/dog-film/relaxation instructions), + 5 min after the film, + 10 min after the film, + 15 min after the film (end of dog intervention/dog-film/relaxation)].

##### Electrocardiogram (ECG)

To measure heart rate in response to the video a standard lead-II ECG with two electrodes was used to collect a raw ECG signal with an ActiveTwo amplifier (BioSemi, Amsterdam, Netherlands) at a sampling rate of 2048 Hz. R-waves were automatically identified by ANSLAB (Wilhelm and Peyk, 2012), edited manually for artifacts, false positives or non-recognized R-waves, and were transformed into instantaneous heart rate. Heart rate was measured constantly during the 5 min baseline period, during the 11 min traumatic film clip and during the 15 min dog intervention.

##### Cortisol

Participants provided six saliva samples for cortisol assessment [before the trauma-film, after the trauma-film, after the dog intervention or control conditions (+15 min), + 30 min, + 45 min, + 60 min]. Cortisol data was collected using Salivette tubes (Sarstedt). The participant first placed a cotton swab provided in each Salivette tube into their mouth and gently chewed on it for about 1 min. The swab was then placed back in the tube. Tubes were kept at −20°C until analysis. Saliva cortisol was analyzed at the cortisol laboratory of the University of Trier, Germany. After thawing the saliva samples for biochemical analysis, the fraction of free cortisol in saliva was determined using a time-resolved immunoassay with fluorometric detection, as described in detail elsewhere (Dressendorfer et al., 1992). For each participant, the area under the curve with respect to increase (AUC_I_) was calculated (Pruessner et al., 2003). The AUC_I_ reflects the increase and decrease of cortisol levels over the entire sampling time and takes into account individual differences in initial cortisol levels.

#### Demographic Data

Demographic data were assessed with an online-questionnaire which was sent to the participants via e-mail after the initial telephone interview. It assessed the age of the participant, trait anxiety (STAI-T), symptoms of depression (BDI-II) and participants’ attitude toward animals (Pet Attitude Scale).

##### STAI-T

Trait anxiety was assessed with the German version of the STAI-T (Laux et al., 1981). The STAI-T contains 20 items measuring trait anxiety. Participants are asked to rate items on a scale from 1 to 4, where ‘1 = not at all’ and ‘4 = totally agree.’ The score for the STAI-T scale ranges from 20 to 80, with 20 indicating a very low and 80 a very high trait anxiety level. As described above for the STAI-S version, reliability and validity have been shown to be high.

##### Pet Attitude Scale

The Pet Attitude Scale is an 18-item scale measuring the general attitude toward pets (Morovati et al., 2008). Items are rated on a seven-point scale (from ‘1 = not at all’ to ‘7 = totally agree’). Participants can reach a maximal score of 126 and a minimal score of 18 points. The scale has been shown to have good psychometric properties.

##### Beck Depression Inventory (BDI-II)

To measure depression symptoms for the last 2 weeks we used the German version of the BDI-II (Hautzinger et al., 1995). Participants can reach scores from 0 to 63 with higher scores indicating more depressive symptoms. A score above 17 is considered to be clinically relevant.

### Experimental Groups

#### Dog Group

Participants in the “dog group” interacted with a dog for 15 min after the traumatic film clip. Dogs were friendly-looking trained therapy dogs of various breeds (Border Collie, Labrador Retriever, Rough Collie, Goldendoodle). All dogs were trained at the same therapy dog training center (Therapiehundezentrum Saar, Mandelbachtal, Germany). The dogs were allowed to move freely within the experimental room. Participants had the opportunity to feed the dog a treat and were encouraged to pet the dog. Video analyses showed that dogs were close to the participants and stroked by them for the majority of the 15-min intervention.

#### Dog-Film Group

Participants in the “dog-film group” watched a 15-min film clip of a person interacting with one of the therapy dogs for 15 min. The clip was filmed in the same laboratory that the participant was sitting in. It was recorded from the perspective of the person interacting with the dog, e.g., participants saw a hand stroking and interacting with the dog. The dog moved freely within the laboratory room. Thus, participants in the “dog-film group” had approximately the same visual input as participants in the “dog group,” but they had no actual contact with the dog.

#### Alone Group

After the trauma-film had ended, participants in the “alone group” were instructed on the screen to relax for 15 min.

### Procedure and Design

The study took place at the laboratories of the Department of Clinical Psychology and Psychotherapy of Saarland University. Participation included two appointments: an initial telephone interview clarifying study eligibility and the actual experimental session. Participants were assigned to one of the three groups (“dog group,” “dog-film group,” “alone group”) by an independent person, who was not member of the research team, according to a computer-generated randomization list.

#### Screening Interview and Online Questionnaire

If participants fulfilled the inclusion criteria in a short telephone interview, a link to the online-questionnaire on SoSciSurvey (Leiner, 2014) assessing demographic data, trait anxiety, depressive symptoms, and attitude toward pets was sent to them. After participants had filled out the online-questionnaire, the experimenter contacted participants again to schedule an appointment for the experimental session.

#### Experimental Session

Upon arrival at the laboratory, participants received an information sheet, informing them about the study’s procedure and goals. Afterward, exclusion criteria were examined, and participants provided written informed consent.

Subsequently, participants were either briefly introduced to the dog (“dog group”) or had a short interaction with the experimenter (other two groups). Then participants were led to the experimental room and the electrodes for physiological measurements (ECG, BP) were attached (see section “Physiological Stress Measurement”). They were then asked to complete the PANAS and the STAI-S questionnaire and to provide their first saliva sample (pre-film measurements). After completing the questionnaires, the experimental procedure began. Participants were told via computer screen that a 5-min-baseline measurement of physiological data would be collected and that they should sit relaxed and look at the screen. After the baseline measurement, participants were informed that they would now be shown the traumatic film and that they should constantly watch the screen and imagine they were an eyewitness of what was happening. Furthermore, on-screen information reminded them that they could withdraw from the experiment at any time if they decided to. Participants then started the film by pressing the “space” button. Physiological data was measured throughout the video. After the traumatic film clip had ended, participants were asked to fill out the PANAS and the STAI-S and to provide the second saliva sample (post-film). Afterward, the dog was brought into the room (“dog group”), the dog-film was started or on-screen instructions told the participants to relax. In all groups, the experimenter entered the room after the traumatic film clip ended, prior to any dog intervention or instructions, asked the participant to take off the headphones and gave instructions regarding the subsequent course of the experiment. This was done to ensure that any differences between the “dog group” and the other two groups would not be due to the contact to the experimenter in the “dog group”. Physiological measurements continued during the 15-min intervention period. After 15 min had passed, the dog was removed from the laboratory and participants were asked to provide another saliva sample and filled out the PANAS and the STAI-S again (post-dog intervention). Then the electrodes were removed and participants were led to another room, where they provided three more saliva samples (30, 45, and 60 min after the trauma-film). During the waiting time, participants were allowed to read magazines. After providing the last saliva sample, participants received 20 Euro for their participation.

### Statistical Analysis

All statistical analyses were conducted using IBM statistics SPSS version 24 (IBM Corp., 2016). Data were analyzed by χ^2^-tests, *t*-tests and mixed design MANOVAs, with the alpha level set to *p* < 0.05. Effect sizes are reported as partial η^2^. To further examine significant interaction effects, we calculated differences from pre- to post-dog intervention and analyzed them using a MANOVA with group as between-subject factor. Unless otherwise stated, all analyses using the factor “group” include all experimental groups (“dog group”, “dog-film group”, and “alone group”). To assess statistical power, we used G^∗^Power (Faul et al., 2009) to calculate *post hoc* power analyses for the relevant time × group interactions.

## Results

### Participant Characteristics

**Table 1** shows that the groups did not differ with respect to age [*F*(2,59) = 0.30, *p* = 0.745], trait anxiety [*F*(2,59) = 0.15, *p* = 0.863], depressive symptoms [*F*(2,59) = 0.12, *p* = 0.890], and their attitude toward pets [*F*(2,59) = 0.42, *p* = 0.660]. Moreover, the experimental groups did not differ concerning the proportion of participants owning a pet [χ^2^(2) = 2.92, *p* = 0.334].

**Table 1:** Means and standard deviations (in brackets) of all experimental groups.

	Dog group (*n* = 20)	Dog-film group (*n* = 20)	Alone group (*n* = 20)	*F*(2,59)	*p*
Age	22.95 (2.87)	22.25 (3.32)	22.65 (2.39)	0.30	0.745
STAI-T	34.15 (7.99)	33.65 (6.50)	34.85 (6.48)	0.15	0.863
BDI-II	4.70 (4.97)	4.90 (4.91)	4.25 (2.79)	0.12	0.890
Pet Attitude Scale	75.95 (7.57)	74.05 (10.71)	73.30 (9.76)	0.42	0.660

### Manipulation Check: Trauma Film Paradigm

As an analog of traumatic experiences, the trauma-film should lead to a significant increase in state anxiety and NA while reported PA should decrease. Further, physiological stress measurements should reflect the stress reaction.

#### Subjective Stress

A MANOVA with time (pre-film, post-film) and group as independent variables and the dependent variables state anxiety, negative, and positive affect showed a significant increase of state anxiety as measured by STAI-S after the film [*F*(1,57) = 122.45, *p* < 0.001, η^2^ = 0.68], which was not moderated by the group allocation [*F*(2,57) = 1.72, *p* = 0.188, η^2^ = 0.06]. For the PANAS ratings the MANOVA also showed a significant increase in NA [*F*(1,57) = 243.32, *p* < 0.001, η^2^ = 0.81] and a decrease of PA [*F*(1,57) = 143,94, *p* < 0.001, η^2^ = 0.72] after the trauma-film. None of these effects was moderated by the experimental group [NA: *F*(2,57) = 0.94, *p* = 0.398, η^2^ = 0.03; PA: *F*(2,57) = 0.18, *p* = 0.837, η^2^ = 0.01] indicating that all experimental groups exhibited a comparable stress reaction to the “traumatic” film clip.

### Physiological Stress Measurements

#### Blood Pressure

A MANOVA with time (pre-film, during film) and group as independent variables and diastolic and systolic BP as dependent variable was calculated. There was a significant effect of time [*Pillai-Spur* = 0.81, *F*(2,56) = 67.98, *p* < 0.001, η^2^ = 0.71] and no effects for group [*Pillai-Spur* = 0.06, *F*(4,114) = 0.90, *p* = 0.455, η^2^ = 0.03] or the group × time interaction [*Pillai-Spur* = 0.05, *F*(4,114) = 0.72, *p* = 0.578, η^2^ = 0.03]. Univariate analyses showed that systolic [*F*(1,57) = 85.61, *p* < 0.001, η^2^ = 0.60] as well as diastolic BP [*F*(1,57) = 112.77, *p* < 0.001, η^2^ = 0.66] were significantly increased after the trauma-film.

#### Electrocardiogram

A mixed MANOVA with time (pre-film, during film) and group as independent factors and HR as dependent variable showed a significant increase in HR [*Pillai-Spur* = 0.30, *F*(1,57) = 24.48, *p* < 0.001, η^2^ = 0.30] after the film, which was not influence by group [*Pillai-Spur* = 0.01, *F*(2,57) = 0.22, *p* = 0.804, η^2^ = 0.01].

### Manipulation Check Trauma Induction

Analogous to traumatic experiences the trauma-film should provoke intrusive memories and thoughts of the trauma-film. Intrusion and thought frequency and distress related to the trauma-film were calculated from the 4 days intrusion diaries. Participants reported on average 3.47 (*SD* = 4.42) intrusive memories of the trauma-film which significantly differed from zero [*t*(56) = 5.93, *p* < 0.001]. The mean distress of the intrusive memories was rated as 5.05 (*SD* = 2.62) on a scale from 0 to 10, also significantly different from zero [*t*(39) = 12.19, *p* < 0.001]. Moreover, participants reported an average of 5.93 (*SD* = 4.84) thoughts of the “traumatic” film, which were also medium distressing (*M* = 5.67, *SD* = 2.92). The frequency of thoughts [*t*(56) = 9.24, *p* < 0.001] and their distress [*t*(56) = 14.66, *p* < 0.001] were also significantly different from zero.

### Group Differences in Subjective Stress

**FIGURE 1:**
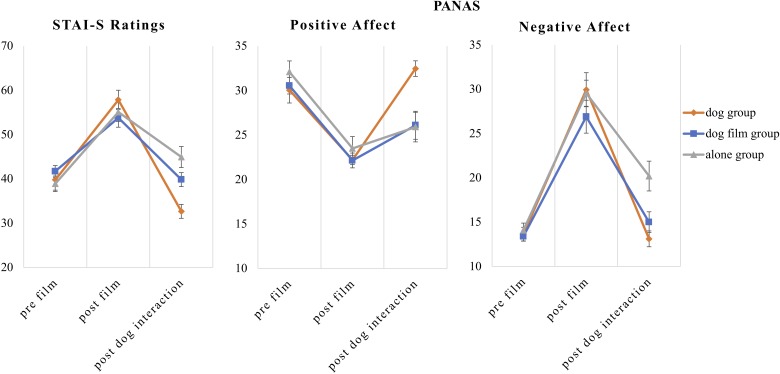
Subjective ratings on state anxiety and positive and negative affect. Error bars indicate SEM.

**Figure 1** shows the subjective ratings of state anxiety and positive and negative affect in all experimental groups. A MANOVA with group and time (pre-film, post-film, post-dog intervention) as independent variables and state anxiety and positive and negative affect as dependent variables showed a significant effect of time [*Pillai-Spur* = 0.87, *F*(6,51) = 56.18, *p* < 0.001, η^2^ = 0.87], no effect of group [*Pillai-Spur* = 0.13, *F*(6,110) = 1.31, *p* = 0.257, η^2^ = 0.07], but a significant time × group interaction [*Pillai-Spur* = 0.52, *F*(12,104) = 3.08, *p* = 0.001, η^2^ = 0.26]. To further investigate the time × group interaction, we calculated differences in state anxiety and positive and negative affect from after the film (prior to the dog intervention) to after the dog intervention (or the dog-film or time alone in the control groups, respectively). These difference scores were compared between the groups using a MANOVA with group as the independent variable. Helmert contrasts which compared the “dog group” to both control groups showed a significantly greater decrease in state anxiety [*t*(56) = −4.85, *p* < 0.001] and NA [*t*(56) = −3.06, *p* = 0.003] as well as a significantly stronger increase of PA [*t*(56) = 5.04, *p* < 0.001] for the “dog group.” There were no significant differences between the two control groups with respect to state anxiety [*t*(56) = −1.24, *p* = 0.221], NA [*t*(56) = −1.11, *p* = 0.273], and PA [*t*(56) = 0.97, *p* = 0.336].

### Group Differences in Physiological Stress Measurements

#### Blood Pressure

To examine group differences in BP reaction we calculated a MANOVA with time (pre-film, during film, start of post-film intervention and three measurements every 5 min during the intervention) and group as independent variables, and diastolic, and systolic BP as dependent variables (see **Table 2** for means and standard deviations of all physiological parameters). There was a significant effect of time [*Pillai-Spur* = 0.74, *F*(10,48) = 48.00, *p* < 0.001, η^2^ = 0.74] and a significant time × group interaction [*Pillai-Spur* = 0.55, *F*(20,98) = 123.26, *p* = 0.024, η^2^ = 0.28]. Contrary to our expectations, both control groups showed a stronger and continued diastolic and systolic BP decrease than the “dog group.” We calculated diastolic and systolic BP changes as the difference between BP during the film and after the dog intervention. While both control groups did not differ significantly, the “dog group” showed a significantly smaller BP decrease [*t*(57) = −2.63, *p* = 0.011].

**Table 2:** Means and standard deviations (in brackets) of all physiological measures per experimental groups.

	Time	Dog group (*n* = 20)		Dog-film group (*n* = 20)		Alone group (*n* = 20)	
Blood pressure (diastolic, systolic)	Pre-film	67.05 (6.98)	113.15 (8.46)	67.05 (7.54)	113.05 (9.40)	70.85 (7.85)	116.65 (10.72)
	During film	74.70 (5.45)	122.00 (10.54)	72.40 (7.82)	121.40 (12.47)	76.80 (8.34)	124.90 (9.95)
	Begin of dog intervention	75.50 (10.56)	129.70 (16.87)	69.85 (9.78)	115.15 (14.29)	74.15 (8.36)	121.80 (14.20)
	5 min	75.75 (6.87)	120.95 (9.71)	68.70 (7.27)	111.65 (9.77)	70.80 (8.12)	116.00 (11.01)
	10 min	75.30 (6.76)	118.90 (9.64)	68.85 (7.94)	111.35 (9.84)	70.70 (9.03)	116.40 (10.88)
	15 min	75.30 (12.57)	120.15 (12.96)	68.35 (7.05)	112.60 (11.67)	69.95 (8.72)	114.95 (9.25)

Cortisol (nmol/l)	Pre-film	3.41 (2.06)		3.79 (2.89)		3.12 (1.49)	
	Post-film	3.40 (1.92)		4.14 (3.97)		3.21 (1.70)	
	15 min	4.60 (3.34)		5.84 (4.81)		5.33 (4.02)	
	30 min	3.93 (2.46)		5.47 (5.51)		4.54 (3.52)	
	45 min	3.63 (2.39)		5.27 (6.16)		4.19 (2.75)	
	60 min	3.31 (1.86)		4.55 (5.01)		3.66 (2.35)	

ECG	Pre-film	73.96 (10.44)		80.98 (15.19)		79.11 (11.06)	
	During film	80.25 (14.10)		86.77 (19.91)		86.84 (15.73)	
	After dog intervention	78.19 (8.90)		75.15 (10.40)		75.29 (11.11)	

#### Cortisol

**FIGURE 2:**
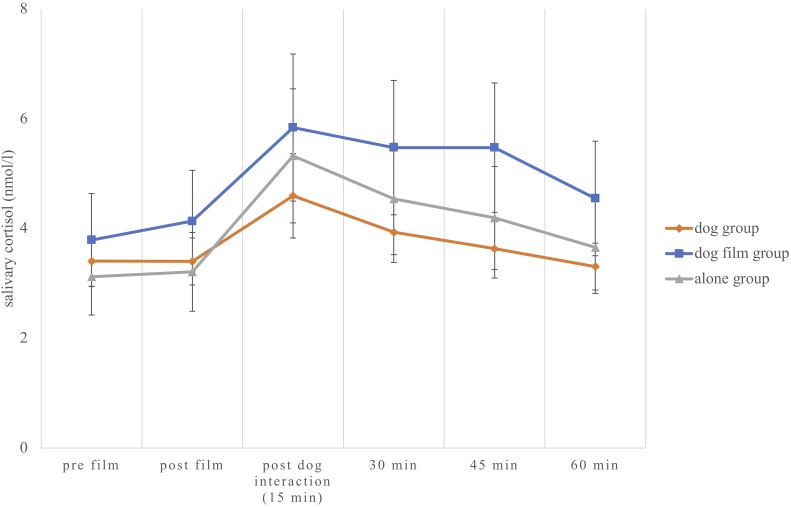
Cortisol response for all experimental groups. Error bars indicate SEM.

We calculated a mixed MANOVA with time (pre-film, post-film, start of post-film intervention and measures after 15, 30, and 60 min), group as between-subject factor and Salivary cortisol as dependent variable (see **Figure 2**). There was a significant effect of time [*Pillai-Spur* = 0.39, *F*(5,52) = 6.71, *p* < 0.001, η^2^ = 0.39], but no significant time × group interaction [*Pillai-Spur* = 0.08, *F*(10,106) = 0.42, *p* = 0.933, η^2^ = 0.04]. We calculated the AUC_I_ and found no significant differences between the groups [AUC_I_: *F*(2,56) = 0.72, *p* = 0.489, η^2^ = 0.03], indicating no differences in cortisol reaction.

#### Electrocardiogram

Group differences in HR were examined using a mixed MANOVA with time (pre-film, during the film, and after the dog intervention) and group as independent variables and HR as dependent variable. There were significant effects of time [*Pillai-Spur* = 0.33, *F*(2,55) = 13.47, *p* < 0.001, η^2^ = 0.33], time × group interaction [*Pillai-Spur* = 0.35, *F*(4,112) = 5.94, *p* < 0.001, η^2^ = 0.18], and no effect of group [*F*(2,56) = 0.46, *p* = 0.631, η^2^ = 0.02]. To further analyze changes during the dog intervention, we calculated the difference of HR during the film to post-dog intervention. Both control groups did not differ significantly from another [*t*(56) = 0.02, *p* = 0.983], but from the “dog group” [*t*(56) = −2.73, *p* = 0.008]. As in BP, the “dog group” showed a smaller decrease in HR from film to post-dog intervention.

### Group Differences in Intrusion Frequency and Intrusion Distress

**FIGURE 3:**
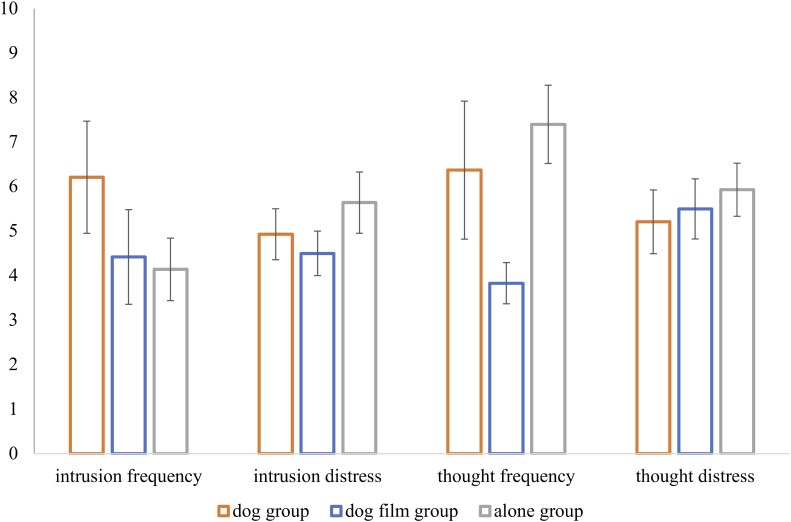
Intrusion frequency and distress per experimental group. Error bars indicate SEM.

Intrusion frequency and distress were compared between the groups using a MANOVA with group as independent variable and intrusion frequency and distress as dependent variables (see **Figure 3**). There was no significant effect of group [*Pillai-Spur* = 0.08, *F*(4,74) = 81.23, *p* = 0.538, η^2^ = 0.04]. The same analysis was applied separately to thought frequency and distress. Concordantly, there was no effect of group [*Pillai-Spur* = 0.13, *F*(4,108) = 1.90, *p* = 0.116, η^2^ = 0.07].

### Power Analyses

*Post hoc* power analyses based on the observed effect sizes, the alpha level of 0.05, and the sample size revealed sufficient statistical power for the time × group interaction for subjective stress ratings (1- *β* = 1.00), blood pressure (1- *β* = 0.98), ECG (1- *β* = 1.00) as well as for cortisol reactivity (1- *β* = 1.00). However, regarding the analyses for intrusion frequency and distress the statistical power was given the small effect insufficient (1- *β* = 0.37). To a smaller extend the same applied to thought frequency and distress (1- *β* = 0.61).

## Discussion

The present study aimed to examine if a therapy dog intervention after an analog “traumatic” event reduces stress and anxiety as well as intrusive memories that emerge as response to the “traumatic” event. The group that received a post-trauma dog intervention showed lower subjectively experienced anxiety (STAI-S) and less NA as well as more PA compared to the two control groups. Contrary to our expectations, the “dog group” showed a smaller decrease in physiologically measured stress levels (blood pressure and heart rate) than the other two groups. Furthermore, we did not find any differences in the cortisol response between the three groups. Finally, the therapy dog intervention did not influence frequency or distress of intrusions and thoughts of the traumatic film.

Our findings are in line with previous studies showing stress and anxiety reducing effects of dogs in stressful situations and with a small number of clinical studies showing that animal-assisted interventions have beneficial effects on traumatized individuals (for a review see O’Haire et al., 2015; O’Haire and Rodriguez, 2018).

As expected, the traumatic film clip led to a significant increase in heart rate and blood pressure. However, contrary to our hypothesis, the dog intervention did not lead to a stronger decrease in these measures as compared to the two control groups. While most studies show that the presence of friendly animals can effectively reduce heart rate and blood pressure during and after a stressor (Beetz et al., 2012b), there are also studies failing to find an effect of an animal intervention on physiological stress measures (e.g., Straatman et al., 1997; Lass-Hennemann et al., 2014). This might have several explanations: in the current study the interaction with the unknown and friendly dog in the experimental room might have acted as a positive stressor causing physiological arousal itself. Another explanation for the lack of a physiological effect may be that participants in the “dog group” exhibited more movement (stroking the dog, turning around to watch the dog) than the other two control groups, thereby inducing physiological arousal. In line with this assumption, Demello (1999) showed that heart rate decreased most during a stressful situation when the pet was present, but no tactile contact was allowed. Further, Grossberg and Alf (1985) showed that heart rate and blood pressure were decreased in contact with a pet in comparison to chatting and reading, but not to rest. In both of our control groups, participants were resting to a certain degree (either watching a film clip or relaxing), which might explain why we did not find the expected results. In this regard, the instruction to relax in the “alone group,” which was actually chosen to control for unspecific effects of relaxation and to investigate the add-on effect of a therapy dog, might have motivated some participants to apply specific relaxation techniques that could have influenced physiological stress levels in terms of a faster decrease. Good control conditions for animal-assisted studies are a topic of constant debate. With our “dog-film group” we established a control group, which realized approximately the same visual input as in the “dog group” and which had the potential to elicit PA in the participants (e.g., Alvarado, 1997) without actual animal contact. Nevertheless, future studies should try to establish a control group in which participants show comparable movement to stroking and interacting with a dog, without actual interaction with a real dog in order to control for the factor “movement” on physiological stress measures.

With respect to relevant control conditions, one might notice a communality between the current study and other studies investigating the influence of post-traumatic interference on symptoms of post-traumatic stress in analog (Holmes et al., 2009) and – however, to date preliminary – in clinical settings (Horsch et al., 2017). These studies found intrusions to be decreased in participants playing the visuospatial demanding computer game “Tetris” after being exposed to an (analog) traumatic event. Even not being a visuospatial task, the dog interaction in the current study could also have acted as such a beneficial interference. However, this explanation should also apply to the “dog-film group” and the pattern of results showing effects on subjective ratings does not correspondent to the findings on post-traumatic interference that mainly report effects on intrusion measures. Nevertheless, further studies could include another control group that investigates the effects of other interfering tasks.

Furthermore, it has to be noted that our study was not designed to investigate possible modes of action by which an interaction with a therapy dog reduces subjective stress levels. Thus, we can only speculate on how our therapy dogs reduced subjective stress and anxiety in our participants. Distraction from the traumatic content during the dog interaction might be one explanation (Gocheva et al., 2018). Another explanation might be a stronger activation of the oxytocin system during the dog interaction as compared to the control conditions (Beetz et al., 2012b). There is a general lack of studies investigating modes of action of animal-assisted interventions and future studies should aim to close this research gap.

Moreover, we assessed frequency and distress of intrusions and thoughts as analog PTSD symptoms in our study and failed to find an influence of the therapy dog intervention on intrusion and thought measures. However, even though intrusions are considered to be one cardinal symptom of PTSD (Friedman et al., 2011), other symptoms such as hyperarousal or changes in cognitions and mood (American Psychiatric Association [APA], 2013) might be more responsive to a therapy dog intervention. To date, most studies assessing the effects of animal-assisted interventions on PTSD assessed general PTSD symptom severity using questionnaires such as the PCL-5 (Blevins et al., 2015) or the Impact of Event Scale (Weiss, 2007). In our opinion, it is important to look at the different PTSD symptoms in more detail in order to discover which PTSD symptoms are most likely to be affected by animal-assisted interventions.

Our experiment was designed as an analog situation for AACR. However, in AACR trained dog/handler teams interact with the traumatized individuals. In our study, we investigated only part of this, namely the dog component. Thus, we cannot draw any conclusions regarding the influence the handler has on the individuals’ stress reaction. It would be interesting to investigate the additive influence of these different parameters by manipulating them (dog alone/dog and handler/handler alone). However, it has to be mentioned that many of the handlers in AACR are volunteers without any psychological background, which may limit the positive impact of the handler within these interventions. Additionally, the timing of the intervention might be crucial. In our study, the “therapy dog intervention” started immediately after the “traumatic” event. Of course, immediate intervention is not feasible after real traumatic events. Moreover, it is also conceivable that immediate action might also be detrimental. In the current study the dog intervention took 15 min which might have been too short to influence post-traumatic stress symptoms like intrusions over a longer period of time. Thus, future studies should have a closer look at the timing, duration, and frequency of animal-assisted interventions after trauma.

### Limitations

One important limitation of our study is that we cannot directly transfer our findings to real traumatic situations. Traumatic events that cause PTSD, provoke different reactions concerning stress and anxiety levels. Also, post-traumatic cognitive processes may be different, not only from a quantitative but also from a qualitative perspective which may in turn limit the ecological validity of analog studies (Vervliet and Raes, 2013) using the trauma-film paradigm. However, this paradigm is the best experimental tool available to mimic stress and anxiety responses to traumatic events (James et al., 2016). Experimental studies allowing for high experimental control are needed to investigate underlying processes and mechanisms in responses to traumatic stressors as well as in PTSD development and course. Nonetheless, clinical replications in PTSD samples investigating the effect of AACR versus psychological first aid versus no intervention after trauma exposure are necessary. Such studies should particularly investigate effects on intrusion frequency and distress as highly clinically relevant symptoms that could not be investigated with sufficient statistical power in the current analog study. A second limitation of our study is that we only assessed female participants who used oral contraceptives. Thus, our data might not be generalizable to non-users of oral contraceptives and men, which should be investigated in further studies.

Another limitation of our study is that positive and negative affect as well as anxiety levels were only assessed during the experimental session. Further studies should assess these measures over a longer period of time to investigate the temporal stability of differences following to the therapy dog intervention.

## Conclusion

The present study extends the knowledge about the effects of therapy dogs on traumatized individuals showing that a therapy dog intervention reduces subjective stress and anxiety after (analog) “traumatic” events. Our study is the first to demonstrate that AACR may be beneficial for traumatized individuals by reducing acute stress and anxiety symptoms.

## Data Availability

The raw data supporting the conclusions of this manuscript will be made available by the authors, without undue reservation, to any qualified researcher.

## Ethics Statement

This study was carried out in accordance with the recommendations of the social and societal ethics committee of the Saarland University. The protocol was approved by the social and societal ethics committee of the Saarland University (15-3). All subjects gave written informed consent in accordance with the Declaration of Helsinki.

## Author Contributions

JL-H designed the study, interpreted the results of all data analyses and drafted the manuscript. SS analyzed the data, helped in interpreting the results, and drafting parts of the manuscript. SR supported the study design process, the data collection and gave feedback during the manuscript writing. EH and MS both contributed to the study design, collected the data and provided an aggregated version of the data. TM contributed to the study design and supported data interpretation and manuscript writing.

## Conflict of Interest Statement

The authors declare that the research was conducted in the absence of any commercial or financial relationships that could be construed as a potential conflict of interest.

## Abbreviations

AACR: animal assisted crisis response
AUC: area under the curve
BDI-II: Beck Depression Inventory II
BP: blood pressure
ECG: electrocardiogram
NA: negative affect
PA: positive affect
PANAS: Positive and Negative Affect Schedule
PCL-5: PTSD Checklist for DSM-5
PTSD: post-traumatic stress disorder
STAI: State-Trait-Anxiety-Inventory.
